# Sex differences in the association between repetitive negative thinking and neurofilament light

**DOI:** 10.1038/s44184-024-00093-8

**Published:** 2024-11-11

**Authors:** Yolanda Lau, Amit Bansal, Cassandre Palix, Harriet Demnitz-King, Miranka Wirth, Olga Klimecki, Gael Chetelat, Géraldine Poisnel, Natalie L. Marchant, The Medit-Ageing Research Group, Anne Chocat, Fabienne Collette, Vincent De La Sayette, Marion Delarue, Hélène Espérou, Eglantine Ferrand Devouge, Eric Frison, Julie Gonneaud, Frank Jessen, Perla Kaliman, Elizabeth Kuhn, Valérie Lefranc, Antoine Lutz, Valentin Ourry, Anne Quillard, Eric Salmon, Delphine Smagghe, Rhonda Smith, Marco Schlosser, Edelweiss Touron, Cédric Wallet, Tim Whitfield

**Affiliations:** 1 Division of Psychiatry, Faculty of Brain Sciences, University College London, London, UK; 2 Normandy University, UNICAEN, INSERM, U1237, PhIND “Physiopathology and Imaging of Neurological Disorders” NeuroPresage Team, Cyceron, Caen, France; 3 German Center for Neurodegenerative Diseases (DZNE), Dresden, Germany; 4 Developmental Psychology, Friedrich Schiller University Jena, Jena, Germany; 5 Université de Liège, Liège, Belgium; 6 Centre Hospitalier Universitaire de Caen, Caen, France; 7 Pôle de Recherche Clinique, INSERM, Paris, France; 8 Normandie Univ, UNIROUEN, Department of General Practice, Rouen, France; 9 Rouen University Hospital, CIC-CRB 1404 Rouen, France; 10 University Bordeaux, INSERM, Institut Bergonié, CHU Bordeaux, CIC 1401, EUCLID/F-CRIN Clinical Trials Platform, Bordeaux, France; 11 Department of Psychiatry, Medical Faculty, University of Cologne, Cologne, Germany; 12 Universitat Autonoma de Barcelona, Barcelona, Spain; 13 Lyon Neuroscience Research Center, Inserm U1028, CNRS UMR5292, Lyon, France; 14 Normandie Univ, UNICAEN, PSL Université, EPHE, INSERM, U1077, CHU de Caen, GIP Cyceron, NIMH, Caen, France; 15 INSERM Transfert, Paris, France; 16 Minerva Health & Care Communications Ltd, Andover, United Kingdom; 17 Department of Psychology Faculty of Psychology and Educational Sciences University of Geneva, Geneva, Switzerland

**Keywords:** Dementia, Biomarkers, Anxiety, Depression

## Abstract

Emerging evidence suggests that repetitive negative thinking (RNT; i.e., worry and ruminative brooding) is associated with biomarkers of Alzheimer’s disease. Given that women have a greater risk of many neurodegenerative diseases, this study investigated whether worry and brooding are associated with general neurodegeneration and whether associations differ by sex. Exploratory analyses examined whether allostatic load, a marker of chronic stress, mediates any observed relationships. Baseline data from 134 cognitively healthy older adults in the Age-Well clinical trial were utilised. Worry and brooding were assessed using questionnaires. Plasma neurofilament light chain (NfL), a biomarker of neurodegeneration, was quantified using a Meso Scale Discovery assay. We found a positive interaction between brooding and sex on NfL, with higher brooding associated with greater NfL levels in women. No associations were observed between worry/ruminative brooding and allostatic load. These results offer preliminary support that RNT is associated with worse brain health, specifically in women.

## Introduction

In the absence of established disease-modifying treatments for dementia, research has shifted towards identifying potentially modifiable factors that may influence risk^1^, and the mechanisms through which they may act. Brain changes in dementia precede cognitive impairment^2^, allowing us to examine premorbid associations between risk factors and pathology. Repetitive negative thinking (RNT) is a modifiable risk factor of recent interest in dementia^3^, and is a transdiagnostic process that encompasses both future-directed worry and past-directed ruminative brooding^4^. RNT describes the thought process, rather than the content of the thoughts. Although RNT levels are elevated in clinical populations (e.g., depression and anxiety^5^), non-clinical populations also engage in RNT, albeit to a lesser degree^6^.

Whilst psychiatric disorders (e.g., depression^7^, anxiety^8^, post-traumatic stress disorder [PTSD]^9^) have been associated with increased risk of dementia, they have primarily been considered independently. The Cognitive Debt hypothesis proposes that RNT may be a common underlying cognitive mechanism that explains the associations between these disorders and increased Alzheimer’s disease (AD) dementia risk^3^. Recent empirical research provides initial support for the Cognitive Debt hypothesis. Higher levels of RNT have been associated with subjective cognitive decline^10^ and more rapid decline in objective cognitive domains affected early in AD dementia, specifically global cognition, immediate and delayed memory^11^. Further, higher levels of RNT have also been associated with hallmark biomarkers of AD (amyloid and tau [PET imaging])^11^, greater brain age^12^, and altered functional connectivity within brain networks associated with AD^13^. However, further evidence is needed to establish whether RNT is associated with neurodegeneration and dementia risk more broadly, beyond AD.

Neurofilament light chain (NfL) is a cytoskeletal protein within neurons that is released following axonal damage or neuronal degeneration^14^. Studies have shown high correlations between elevated NfL levels and standard measures of neurodegeneration such as measured through magnetic resonance imaging^15,16^, underscoring NfL’s role as a robust surrogate marker of neurodegeneration. A recent meta-analysis found increased levels of NfL in all common neurodegenerative illnesses of advanced age, including AD, Lewy Body dementia, vascular dementia and frontotemporal dementia^17^. Notably, many of these dementia types are not associated with amyloid and tau aggregation. Research has further supported the predictive value of increased NfL levels for the later development of dementia in healthy older adults^18^ and individuals at risk of dementia^19^. Since raised NfL levels are common across different types of dementia, the current study examined the relationship between RNT and NfL to determine whether RNT could be broadly associated with dementia risk.

Women have a greater lifetime risk of developing AD and dementia, thus there is increasing acknowledgement that potential sex and gender differences need to be examined in dementia research^20,21^. There are sex and gender differences in risk factors for AD. For instance, depression (a psychosocial risk factor of dementia^1^) is more prevalent in women than men^22^. There are also sex and gender differences in the progression of cognitive decline and AD, with women having faster deterioration than men^23^. Additionally, there is evidence of sex differences in different inflammatory markers^24^ (increased levels of inflammatory markers have been associated with increased risk of dementia^25^). This highlights the importance of considering sex and gender differences when investigating RNT and neurodegenerative biological markers, and potential pathways of action.

Accumulating evidence provides support for a prospective association between stress and dementia. In addition to the link between PTSD and dementia^9^, recent meta-analyses have shown positive associations between psychological stress, trauma and dementia^26,27^ and revealed that older adults who experienced more stressful life events are at higher risk of developing dementia^28^. Based on existing evidence showing associations between RNT and markers of stress (e.g., cortisol, blood pressure)^29^, one potential mechanism linking RNT with poor brain health could be via a ‘stress pathway’. Longitudinal studies have found that trait rumination (of which brooding is a maladaptive subtype) and trait worry predict the onset of PTSD symptoms after exposure to a traumatic event^30^.

Stress not only has an impact on the brain, but also has an effect on the body more broadly. To describe the biological consequences of chronic stress on the body, Bruce McEwen conceptualised the term allostatic load^31^. The concept of allostatic load is derived from the term *allostasis*, which is an adaptive response that refers to the body’s ability to adapt to the needs of different situations (including activation of the stress response system when under threat)^32^. When chronically stressed over time, the body’s stress system becomes overactivated, which in turn increases allostatic load (a maladaptive response). Higher levels of allostatic load are associated with cognitive decline in older adults^33^, which in turn, has been associated with an increased risk of developing dementia^1^. Biological and physiological markers of stress are common components of an allostatic load index. Cortisol is one marker which has been linked to cognitive decline as part of an index to measure allostatic load^33^. Other components include waist-to-hip ratio, blood pressure, and cholesterol^34^. Allostatic load affects multiple bodily systems, including anthropometric, cardiovascular and respiratory, metabolic, immune, and neuroendocrine systems; therefore comprehensive allostatic load indices include measures from each system.

Extant evidence demonstrates associations between anxiety and depression and increased allostatic load^35^, yet the direct association between RNT and allostatic load is unknown. Studies have however shown associations between state level or induced RNT and some of the biomarkers commonly included in allostatic load composites, for example higher heart rate, cortisol levels, and blood pressure^29^. However, it is unknown whether trait RNT is associated with allostatic load and whether allostatic load could mediate any association observed between RNT and neurodegeneration biomarkers.

We sought to further understand the relationship between RNT and neurodegeneration, and a possible mechanism through which RNT may act. More specifically, we aimed to determine (1) the cross-sectional relationship between two measures of RNT (worry and ruminative brooding) and NfL in cognitively healthy older adults and whether this relationship is moderated by sex. We further sought to understand whether allostatic load mediates this relationship in exploratory analyses. We hypothesised that higher levels of RNT would be associated with greater NfL and that this relationship would be mediated by allostatic load.

## Materials and methods

### Study design

The current study utilised cross-sectional baseline data from the Age-Well randomised clinical trial of the Medit-Ageing European project. Details of the recruitment method and eligibility criteria can be found in the Age-well protocol^36^.

### Participants

137 participants were recruited from the general population (Caen, France), screened, and included in the trial. All participants were required to be aged 65 or over, have at least seven years of education, and be retired for at least one year. They also performed normally on standardised cognitive tests measuring various domains (global cognitive functioning, executive functions, and verbal episodic memory) according to study-specific standards (age, sex, and education level). Detailed descriptions of performance requirements for each test are provided in the Supplementary Material 1. Key exclusion criteria included the presence of major neurological or psychiatric disorder, chronic disease or acute unstable illness that may interfere with cognitive functioning. Further details relating to inclusion and exclusion criteria can be found in the trial protocol paper^36^.

Written informed consent was obtained from all participants. The Age-Well randomized clinical trial was sponsored by the Institut National de la Santé et de la Recherche Médicale (Inserm), approved by the ethics committee (Comité de Protection des Personnes Nord-Ouest III, Caen, France; trial registration number: EudraCT: 2016-002441-36; IDRCB: 2016-A01767-44) and registered on ClinicalTrials.gov (Identifier: NCT02977819).

### Repetitive negative thinking (RNT)

#### Worry

Worry was measured using the self-report 16-item Penn State Worry Questionnaire (PSWQ)^37^. Each item is rated on a 5-point Likert scale, ranging from 1 (“not at all typical of me”) to 5 (“very typical of me”). Total scores range from 16 to 80, with higher scores being indicative of higher levels of worry. Previous research has demonstrated good internal consistency and adequate convergent validity in older adults^38^.

#### Ruminative brooding

Ruminative brooding was measured using the 5-item brooding subscale of the self-report 22-item Ruminative Response Scale (RRS)^39^. Each item is rated on a 4-point Likert scale, ranging from 1 (“almost never”) to 4 (“almost always”). Total scores range from 5 to 20, with higher scores being indicative of higher levels of ruminative brooding. Previous research has demonstrated acceptable internal consistency in older adults^40^.

### Neurofilament Light

Non-fasting blood samples were collected from participants between 7.30 am and 8 am. Samples were drawn from the antecubital vein into a 5 ml EDTA bottle and then, after decantation and collection of plasma, stored in a -80 °C freezer before analysis. Plasma levels of NfL were performed by an ultra-sensitive electrochemiluminescence measurement technique (Meso Scale Discovery, MSD, Rockville, USA) using R-PLEX Human Neurofilament L Antibody Set with reference ranges of 5.5 to 50,000 pg/mL. All analyses took place in the Centre de Ressources Biologiques (CRB) in Caen, France.

### Allostatic load

The allostatic load composite created in the current study was comprised of five sub-categories: anthropometric, cardiovascular and respiratory, metabolic, immune, and neuroendocrine. This composite measure in this study is grounded in a conceptual framework that is based on the idea that chronic stress affects multiple physiological systems, measurable using biomarkers. The selection of individual biomarkers included in this allostatic load composite are frequently used in existing allostatic load studies^41^. Detailed descriptions of the measures included in the composite are provided in the Supplementary Material 2.

Briefly:

*Anthropometric category* included measures of body mass index (BMI) and waist-hip ratio (WHR).

*Cardiovascular and respiratory category* included measures of systolic and diastolic blood pressure (SBP, DBP), pulse pressure, standard deviation of the average heartbeat-to-beat intervals (SDANN) and root mean square of successive differences between normal heartbeats (RMSSD).

*Metabolic category* included measures of plasma insulin, serum triglycerides, high and low-density cholesterol (HDL, LDL) and serum creatinine.

*Immune category* included plasma measures of interleukine-6 (IL-6) and C-reactive protein (CRP).

*Neuroendocrine category* included serum cortisol, dehydroepiandrosterone sulfate (DHEA-S), plasma norepinephrine (NE) and epinephrine (E).

Measures within each sub-category were z-transformed and averaged to create sub-category scores. The average of each of the five sub-categories was then calculated and re-standardised to form the total allostatic load score, resulting in a mean of 0 and a standard deviation of 1. Values for HDL, DHEA-S, SDANN, and RMSSD were reverse-scored before being included in the composites, so that higher scores indicated higher levels of allostatic load. Participants with missing scores on any of the measures were not included in the composite score calculation.

### Covariates for sensitivity analyses

To examine the specific effects of the cognitive component of RNT (i.e., ruminative brooding and worry), we conducted sensitivity analyses controlling for depression (for ruminative brooding) and anxiety (for worry) due to their close association with RNT^42,43^.

#### Depression

Depression was measured using the self-report 15-item Geriatric Depression Scale (GDS-15)^44^. Each item is answered with either “yes” or “no”. Total scores (calculated by summing the number of “yes” responses) range from 0 to 15, with higher scores being indicative of higher levels of depression. Previous research has demonstrated good internal consistency^45^ and validity^44^ in older adults.

#### Anxiety

Anxiety was measured using the 20-item trait anxiety subscale of the State-Trait Anxiety Inventory (STAI-B)^46^. Each item is rated on a 4-point Likert scale, from 1 (“almost never”) to 4 (“almost always”). Total score for STAI-B subscale (calculated by summing all the respective item scores) range from 20 to 80, with higher scores being indicative of higher levels of anxiety. Previous research has demonstrated good internal consistency and validity for older adults^47^.

### Statistical analyses

From the 137 participants enrolled in Age-Well, two participants were excluded from all secondary analyses for not meeting major eligibility criteria as determined by the Trial Steering Committee. One participant had missing data for NfL. This resulted in 134 participants being included in the analyses with NfL. Participants with missing data for at least one of the measures included in the allostatic load composite were excluded in analyses with allostatic load.

Associations between RNT, NfL, allostatic load and potential confounds were investigated using Pearson’s correlations for continuous variables, and t-tests for sex differences.

Prior to performing the planned statistical analyses, assumptions of linear regression were checked. Ruminative brooding was right skewed and was therefore square-root transformed. The assumptions of linearity, homoscedasticity and independence were met for all models, using the square-root transformed ruminative brooding scores. The assumption of normality was not met; however, as the importance of linearity supersedes normality in regression models^48^, we proceeded with using square root-transformed ruminative brooding scores in all analyses.

Separate linear regression analyses were run for each RNT measure (i.e., worry and ruminative brooding) to examine their relationships with NfL. Model 1 was unadjusted, Model 2 adjusted for age, sex and education, and Model 3 examined the (additive) interaction with sex, adjusting for age and education. Sensitivity analyses were run to determine whether any associations remained after adding anxiety to Model 2 and Model 3 when worry was the predictor or depression when ruminative brooding was the predictor. Given the low levels of depressive symptoms observed in the sample, analyses were conducted using depression as a categorical variable (participants with no depressive symptoms [i.e., scores of 0] and participants with any depressive symptoms [i.e., scores of 1 or above])^49^.

Finally, to uncover a potential mechanism linking RNT to NfL, we conducted exploratory analyses to examine the relationship between worry and ruminative brooding and allostatic load in unadjusted and adjusted regressions (i.e., Model 1 and Model 2, as above). Model 3 examined the interaction with sex, adjusting for age and education. In the case of a significant association being observed between worry and/or brooding and NfL, and between worry and/or brooding and allostatic load, a mediation analysis would be conducted to investigate whether allostatic load mediated the relationship between worry and/or brooding and NfL. Meditation analysis is used to understand the mechanism through which an independent variable influences a dependent variable via a mediator variable^50^. The four-step mediation analysis framework developed by Baron and Kenny^51^ was used, with each step needing to be met before proceeding to the next. The first step is to demonstrate that the independent variable (i.e., RNT) significantly affects the dependent variable (i.e., NfL). The second step is to demonstrate that the independent variable significantly affects the mediator (i.e., allostatic load). The third step is to demonstrate that the mediator affects the dependent variable when controlling for the independent variable. The final step is to establish the direct effect of the independent variable on the dependent variable while controlling for the mediator.

Exploratory analyses were conducted to examine the associations between worry and ruminative brooding and each sub-domain of the allostatic load composite.

All analyses were performed with in R (version 4.2.2). Standardised beta coefficients are reported.

## Results

Demographic characteristics are presented in Table 1. Associations between continuous variables are displayed in Table 2. Briefly, worry was associated with anxiety (*r* = 0.62, *p* < 0.001) and depression (*r* = 0.17, *p* = 0.07). Ruminative brooding was associated with anxiety (*r* = 0.51, *p* < 0.001) and depression *(r* = 0.18, *p* = 0.06). NfL was associated with age (*r* = 0.39, *p* < 0.001). Allostatic load was not associated with age (*r* = 0.09, *p* = 0.37), education (*r* = -0.11, *p* = 0.25), depression (*r* = -0.01, *p* = 0.88), or anxiety (*r* = -0.11, *p* = 0.27).

**Table 1:** Participant characteristics (*n* = 134)

Variable	Total	Women (*n* = 82)	Men (*n* = 52)
	Mean or N (SD or %)	Mean or N (SD or %)	Mean or N (SD or %)
**Demographics**			
Age	69.3 (3.8)	69.2 (4.0)	69.3 (4.0)
Women^a^, N, %	82 (61.2)		
Education, years	13.2 (3.1)	12.6 (3.0)	14.1 (3.1)
**RNT**			
Ruminative brooding (RRS-B)^b^	8.1 (2.3)	8.1 (2.2)	8.1 (2.5)
Worry (PSWQ)	41.9 (11.5)	42.4 (11.7)	40.7 (11.2)
**Allostatic load**^**c**^	0.0 (1.0)	0.0 (1.0)	0.0 (1.0)
**NfL** (pg/mL)	19.9 (7.6)	20.0 (8.4)	19.9 (6.2)
**Psychiatric symptoms**			
Anxiety symptoms (STAI-B)	34.6 (7.0)	35.2 (7.0)	33.5 (7.1)
Depressive symptoms (GDS), continuous	1.3 (1.7)	1.6 (2.0)	0.8 (1.2)
Depressive symptoms (GDS), categorical			
Presence of depressive symptoms	57 (32.9%)	27 (33.0%)	30 (58.9%)
No depressive symptoms	76 (57.1%)	55 (67.1%)	21 (41.2%)

Data are presented as mean and standard deviation unless otherwise specified.

*RRS-B* Brooding subscale of the Rumination Response Scale, *PSWQ* Penn State Worry questionnaire, *STAI-B* trait subscale of the State-Trait Anxiety Trait Inventory, *GDS* Geriatric Depression Scale.

^a^Participants self-reported their sex

^b^*N* = 133, ^c^*N* = 111

**Table 2:** Correlation matrix for continuous variables

	Age	Education	Ruminative brooding	Worry	Anxiety symptoms	Depression symptoms	Allostatic load	NfL
**Age**	1.00	-0.21*	-0.08	-0.07	-0.08	-0.09	0.09	0.39*
**Education**		1.00	-0.01	-0.07	-0.05	-0.04	-0.11	0.02
**Ruminative brooding**			1.00	0.49*	0.51*	0.18	-0.04	0.10
**Worry**				1.00	0.62*	0.17	-0.01	0.09
**Anxiety symptoms**					1.00	0.39*	-0.11	0.08
**Depressive symptoms**						1.00	-0.01	0.03
**Allostatic load**							1.00	-0.11
**NfL**								1.00

^*^p < 0.05

Regarding sex differences, there was no difference in NfL levels (*p* = 0.82), worry (*p* = 0.48) or ruminative brooding *(p* = 0.90) between men and women. Allostatic load was higher in men than in women (*p* = 0.02). There were no differences in worry or ruminative brooding between men and women. There was a statistically significant sex difference in depression symptoms, with women (mean = 1.60) having higher levels than men (mean = 0.81) (*p* = 0.004).

### Associations between RNT and NfL

No association was observed between ruminative brooding and NfL in the unadjusted model (Model 1: β = 0.10, 95% confidence interval [CI] -0.08 to 0.27, *p* = 0.268) or adjusted model (Model 2: β = 0.14, 95% CI -0.02 to 0.30, *p* = 0.10).

No association was observed between worry and NfL in the unadjusted model (Model 1: β = 0.10, 95% CI -0.07 to 0.27, *p* = 0.233) or adjusted model (Model 2: β = 0.15, 95% CI -0.01 to 0.31, *p* = 0.07).

**Fig. 1:**
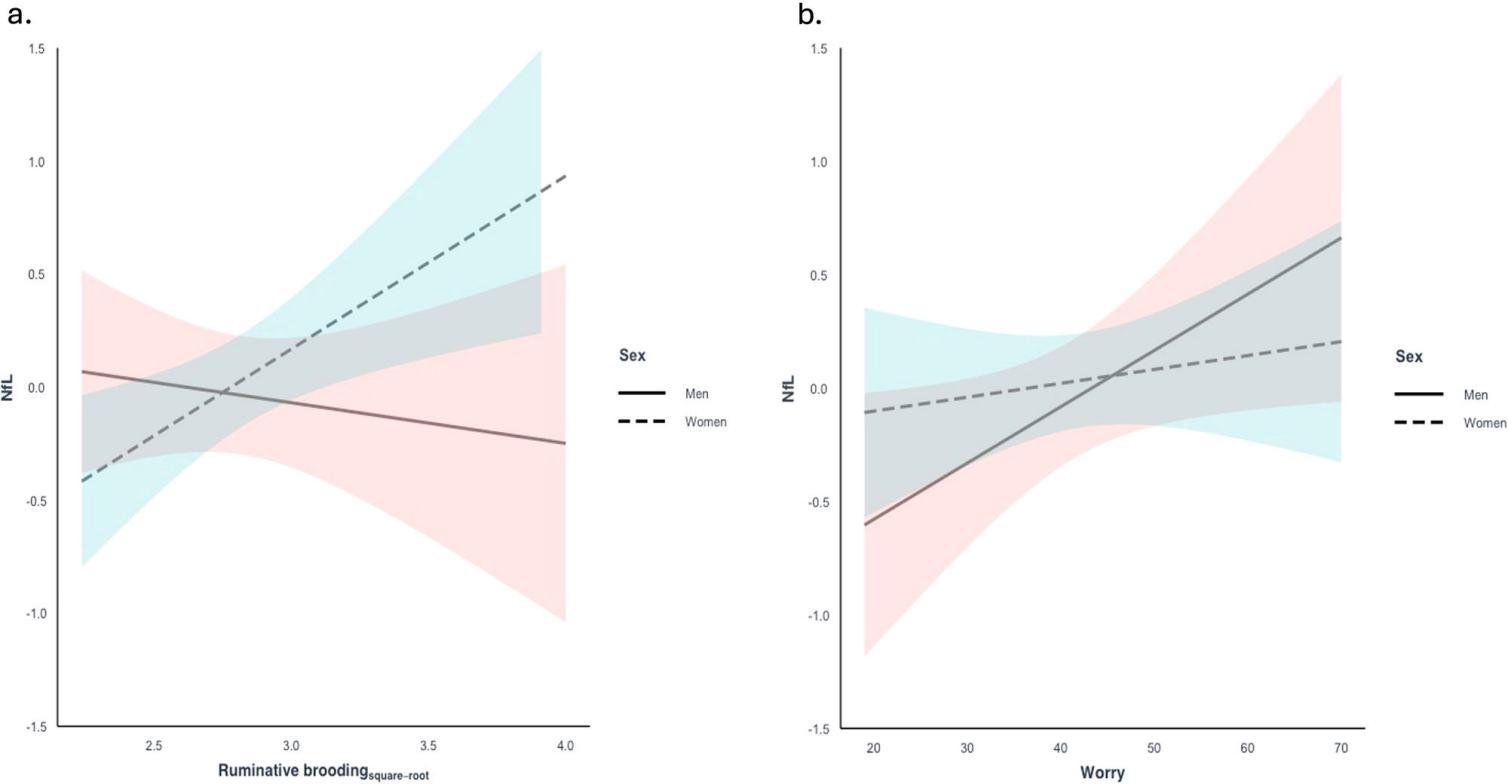
Associations between ruminative brooding, worry, and NfL levels stratified by sex. Associations between **a** ruminative brooding and NfL and **b** worry and NfL, adjusting for age and education, and stratified by sex. A significant interaction was observed between ruminative brooding and sex on NfL. Higher levels of ruminative brooding were associated with elevated NfL levels in women, while no such association was observed in men. No interaction was observed between worry and sex on NfL.

A statistically significant interaction was observed between ruminative brooding and sex on NfL after adjusting for age and education (β = 0.37, 95% CI 0.05 to 0.69, *p* = 0.026 [reference group: women]) (Fig. 1a). In women, after adjusting for age and education, higher levels of ruminative brooding were associated with higher levels of NfL (β = 0.27, 95% CI 0.06 to 0.46, *p* = 0.01). In men, no associations were observed between ruminative brooding and NfL (β = -0.10, 95% CI -0.36 to 0.17, *p* = 0.47). In sensitivity analyses adding depression, results remained unchanged.

No statistically significant interactions were observed between worry and sex on NfL after adjusting for age and education (β = 0.02, 95% CI -0.01 to 0.05, *p* = 0.207 [reference group: women]) (Fig. 1b).

### Associations between RNT and allostatic load

**Fig. 2:**
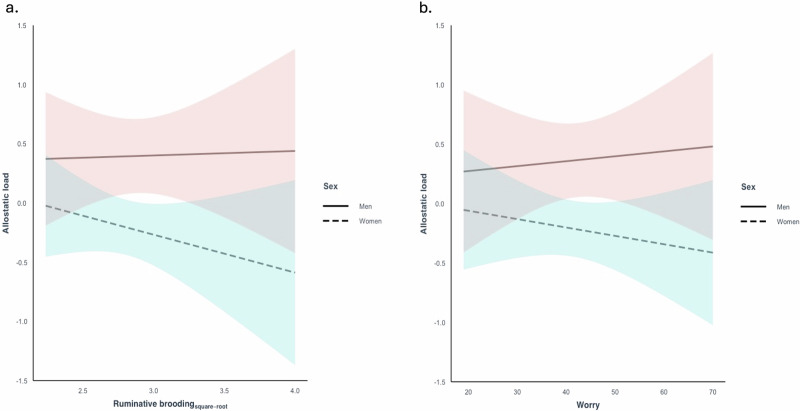
Associations between ruminative brooding, worry, and allostatic load stratified by sex. Associations between **a** ruminative brooding and allostatic load and **b** worry and allostatic load, adjusting for age and education, and stratified by sex. No interaction was observed between ruminative brooding and sex on allostatic load, or between worry and sex on allostatic load.

No association was observed between ruminative brooding and allostatic load in the unadjusted model (Model 1: β = -0.04, 95% CI -0.23 to 0.15, *p* = 0.657) or adjusted model (Model 2: β = -0.06, 95% CI -0.25 to 0.12, *p* = 0.488). No interaction was observed between ruminative brooding and sex on allostatic load after adjusting for age and education (β = 0.14, 95% CI -0.51 to 0.23, *p* = 0.460 [reference group: women]) (Fig. 2a).

No association was observed between worry and allostatic load in the unadjusted model (Model 1: β = -0.01., 95% CI -0.20 to 0.18, *p* = 0.938) or adjusted model (Model 2: β = -0.02, 95% CI -0.002 to 0.33, *p* = 0.30). No interaction was observed between worry and sex on allostatic load after adjusting for age and education (β = 0.02, 95% CI -0.02 to 0.05, *p* = 0.3617 [reference group: women]) (Fig. 2b).

Sensitivity analyses were not conducted because no associations were observed between ruminative brooding or worry and allostatic load.

### Exploratory Analyses

In the first step of mediation analysis, we demonstrated that ruminative brooding affects NfL in women. In the second step, ruminative brooding was not associated with allostatic load and no sex interaction was observed therefore we did not proceed with the mediation analysis. According to the approach outlined by Baron and Kenny, each step builds upon the previous step, therefore, if any of the steps are not met, the process stops^51^.

No associations were observed between ruminative brooding or worry and any of the five allostatic load sub-domains (all *p* values ≥ 0.095, see Supplementary Material 3).

## Discussion

The current study aimed to examine the relationship between RNT (including worry and ruminative brooding) and NfL, a marker of neurodegeneration and dementia risk, and whether this relationship was modulated by sex in cognitively healthy older adults. We further explored the potential mechanism through which RNT may confer increased risk by examining relationships between RNT and a marker of chronic stress, allostatic load. As expected, age was positively associated with NfL. We observed no associations between both measures of RNT and NfL; however, we did observe an interaction between brooding and sex on NfL. Higher levels of brooding were associated with higher levels of NfL in women, while there were no associations observed in men. RNT was not associated with allostatic load in the whole sample or when examining women alone.

Women are at greater risk of developing dementia, and RNT (which includes ruminative brooding and worry) is a common cognitive process that has been associated with multiple psychiatric conditions and is a feature of neuroticism, all of which have been associated with dementia^7–9,52^. Existing literature examining whether sex moderates these associations is conflicting and relatively scarce^53,54^. Therefore, our findings align with existing literature and extends this body of work by showing that a key symptom of these psychiatric conditions (i.e., brooding) is associated with a biomarker of neurodegeneration and dementia (i.e., NfL), specifically in women.

Depression has consistently been associated with increased risk of dementia. Brooding is strongly associated with depression and predicts the onset duration and chronicity of depressed mood^55^. Brooding can be described as repetitive passive judgemental thoughts, including thoughts about depressive symptoms and their possible causes and consequences. The finding that brooding was associated with higher NfL fits with existing research that has reported relationships between clinical depression and NfL^56^, and indeed specifically in older-adult women^57^. Notably our observation of an association between brooding and NfL in women remained after adjusting for depressive symptoms, indicating a specificity of that cognitive process in this relationship. The biological mechanism through which depression may confer this risk may therefore be common to that of brooding. We did not observe the same relationship between worry and NfL. Worry captures the dominant psychological thinking style in Generalised Anxiety Disorder which also has some evidence to suggest a higher risk of later developing dementia^58^. Our findings suggest that whilst both may increase risk, depressive-related brooding may confer a higher risk. This may be related to the ability of brooding to maintain depressive symptoms^59^, there may be a differential consequence of brooding on social functioning similar to that of depression in comparison to anxiety^60^, or the disengagement from problem solving caused by brooding^61^.

Our findings that the relationship between brooding and NfL is stronger in women aligns with existing literature showing sex differences in dementia risk. In addition, sex differences in depression are well established in the literature, depression is more prevalent among women than men^62^. Indeed, we observed higher levels of depression symptoms in women than men in our study. We did not observe a difference in brooding levels between men and women (in contrast to findings from a meta-analysis^63^), which could suggest that women have heightened susceptibility to the adverse effects of brooding in the brain, as measured by NfL, compared to men. In support of this interpretation, depression has been related to hippocampal loss in women only^64,65^. A previous systematic review reported inconsistent findings on the association between RNT and neurodegeneration^66^. One potential explanation for these inconsistencies could be because sex moderates the relationship, and none of the included studies examined sex interactions. Our findings extended this systematic review and show that sex should be examined as a moderator when examining the association between RNT and neurodegeneration. The significance of this finding more broadly is that cognitive decline is associated with higher levels of brain atrophy and later development of dementia, which is why our finding could implicate brooding as a sex and gender-dependent risk factor for dementia^67^. The mechanism of this higher dementia risk in women remains unclear. Evidence, however, has highlighted differential sex stress responses in corticolimbic circuitries, the hypothalamic-pituitary–adrenal axis (HPAA), and the autonomic nervous system (ANS) that may help to explain the different impact of brooding on NfL in women found in our study^68^. It is also possible that there are different sequelae of brooding in women compared to men which leads to increased risk, similar to how there are different presentations of depression in women with them reporting higher hypochondriasis, weight gain, appetite and somatic concerns^69^.

The positive association we observed between brooding and NfL builds on a previous report that RNT is associated with markers of AD^11^. NfL is more broadly related to all-cause dementia and therefore our findings may suggest a wider link between brooding and dementia beyond AD^18^. Given the close relationship between elevated NfL and neuroimaging markers of brain atrophy^67^, our findings reinforce those from a study which observed that brooding was associated with an accelerated brain age (as opposed to chronological age)^12^. In addition to structural alterations, which have been observed across diverse and disparate brain regions^70^, brooding has been associated with functional brain alterations^13,71^. For instance, Schwarz et al. found that higher negative affective burden was associated with lower between-network functional connectivity of default mode network (DMN) and salience network (SAL) nodes^13^. Further, they found that in older adults with subjective cognitive decline (which is a risk factor for dementia^72^), higher negative affect was associated with functional connectivity in networks that are affected early in AD – higher resting-state functional connectivity within DMN (posterior cingulate-to-precuneus) and within SAL (anterior cingulate-to-insula) nodes^13^. None of the above studies examined sex interactions, which will be an important consideration for future work in this area.

We predicted that RNT would be associated with higher levels of allostatic load given previous reports of its association with markers of stress (e.g., cortisol, elevated blood pressure, etc)^29^. Contrary to our predictions, however, we did not find evidence that ruminative brooding or worry were associated with allostatic load in the whole sample or in interaction with sex. Potential explanations for the null results could be due to methodological differences in the assessment of RNT and stress. For example regarding RNT, Ottaviani et al.^29^ found that when RNT was induced (e.g. asking participants to actively worry) or reflected present moment thinking it was associated with markers of stress, whereas our study measured participants’ trait brooding and worry. Regarding stress, their systematic review assessed acute changes in stress markers (i.e., assessed changes in blood pressure, heart rate, cortisol, and/or heart rate variability during RNT or after RNT induction), whereas our study measured cumulative physiological consequences of chronic stress. It could be that an active state of engaging in RNT is associated with an acute biophysiological stress response whereas chronic, trait brooding is not associated with markers of chronic stress (e.g., allostatic load).

This study has several strengths. First, it addresses the call to study sex differences^21^ while assessing associations between RNT and NfL, and between RNT and allostatic load in older adults. Second, this study used separate questionnaires to measure two components of RNT, worry and ruminative brooding. Assessment of worry and ruminative brooding allowed us to examine their distinct and overlapping relationships with NfL. We found a positive association between the brooding component of RNT and NfL in women although no relationship with allostatic load, supporting the idea of a negative thinking style being associated with brain health. Further, this provides support for adopting a more transdiagnostic approach when examining psychological mechanisms associated with the development of dementia. The consequences of these findings are the potential recognition of a modifiable risk factor in an at-risk population (women) that can be targeted through interventions. Finally, the allostatic load composite created in this study includes a wide variety of biomarkers, thus having greater sensitivity and reliability compared to looking at the biomarkers individually.

This study also has limitations. First, participants in the Age-Well trial were highly educated and were relatively healthy (physically and mentally). Therefore, these findings may not be generalisable to the wider older adult population, and this may have resulted in reduced variability in allostatic load levels. Second, there are methodological differences in the construction of the allostatic load measure in the present study compared with other studies in this area. The biomarkers included in allostatic load composites are primarily treated as dichotomous variables (using cut-offs) in most other studies^73^, however, in our study, the biomarkers are treated as continuous variables (z-scores). While we consider it a strength to construct the allostatic load composite as a continuous variable, taking into account the continuous properties of each variable included in the composite and the number of variables in each sub-category, it is possible that there might have been a threshold effect. Utilising cut-offs could have allowed us to detect such a threshold effect. Third, the relatively small total sample size, which is further exacerbated when dividing the sample into men and women, reduces statistical power and increases the likelihood of Type II errors. Whilst we did not observe an association between brooding and NfL in men, this could be due to the absence of a true effect, insufficient power, or the sex imbalance in our sample. Finally, the cross-sectional study design precludes us from determining causality. Whilst we propose that high levels of RNT may result in higher levels of NfL, the opposite could also be true.

Brooding was associated with elevated levels of NFL in women, providing support that RNT is associated with dementia risk in cognitively healthy older adults. Longitudinal studies are needed to establish the directionality of this association. Whether reducing brooding subsequently reduces NfL and dementia risk is still unknown, yet encouragingly prospective cohort studies have shown that psychological interventions that reduced anxiety and depression in older adults (e.g., cognitive behavioural therapy, mindfulness) resulted in reduced incidence of future dementia^74,75^. The recognition of brooding as a modifiable risk factor for dementia is exciting because it would unlock the prosect of public health interventions in a subclinical population of those who do not meet the threshold of any clinical mental health diagnosis. Randomised clinical trials with biomarker assessments will be needed to clarify the causal relationships and mechanisms. In addition, future studies, with more diverse samples, would increase the generalisability of the findings. Furthermore, although this study suggests that the stress-pathway (i.e., allostatic load) may not be the mechanism that links brooding with dementia risk, future studies could investigate whether relationships emerge in a clinical sample or in individuals with high allostatic load.

This is the first study to investigate the relationship between RNT and NfL, and whether allostatic load mediates this relationship, in cognitively healthy older adults. Our study found no evidence that RNT was associated with NfL when considering both sexes together; however upon separating the sample by sex, we found a statistically significant association between brooding and NfL in women. We found no evidence that this association was mediated by allostatic load. These findings contribute to the growing interest in dementia research that emphasises sex-specific risk. Research in this area could inform sex-specific dementia prevention and intervention. Elucidating the mechanism(s) that underlie the association between psychological processes and dementia risk will be critical in helping to inform interventions aimed at reducing dementia risk.

## Supplementary information

Supplementary materials

## Data Availability

The data underlying this manuscript are made available on request following a formal data sharing agreement and approval by the consortium and executive committee (https://silversantestudy.eu/2020/09/25/data-sharing). The Material can be mobilized, under the conditions and modalities defined in the Medit-Ageing Charter by any research team belonging to an Academic, for carrying out a scientific research project relating to the scientific theme of mental health and well-being in older people. The Material may also be mobilized by non-academic third parties, under conditions, in particular financial, which will be established by separate agreement between Inserm and by the said third party. Data sharing policies described in the Medit-Ageing charter are in compliance with our ethics approval and guidelines from our funding body.
