# Author Correction: Sex differences in the association between repetitive negative thinking and neurofilament light

**DOI:** 10.1038/s44184-025-00116-y

**Published:** 2025-01-18

**Authors:** Yolanda Lau, Amit Bansal, Cassandre Palix, Harriet Demnitz-King, Miranka Wirth, Olga Klimecki, Gael Chetelat, Géraldine Poisnel, Natalie L. Marchant, The Medit-Ageing Research Group, Anne Chocat, Fabienne Collette, Vincent De La Sayette, Marion Delarue, Hélène Espérou, Eglantine Ferrand Devouge, Eric Frison, Julie Gonneaud, Frank Jessen, Perla Kaliman, Elizabeth Kuhn, Valérie Lefranc, Antoine Lutz, Valentin Ourry, Anne Quillard, Eric Salmon, Delphine Smagghe, Rhonda Smith, Marco Schlosser, Edelweiss Touron, Cédric Wallet, Tim Whitfield

**Affiliations:** 1 Division of Psychiatry, Faculty of Brain Sciences, University College London, London, UK; 2 Normandy University, UNICAEN, INSERM, U1237, PhIND “Physiopathology and Imaging of Neurological Disorders” NeuroPresage Team, Cyceron, Caen, France; 3 German Center for Neurodegenerative Diseases (DZNE), Dresden, Germany; 4 Developmental Psychology, Friedrich Schiller University Jena, Jena, Germany; 5 Université de Liège, Liège, Belgium; 6 Centre Hospitalier Universitaire de Caen, Caen, France; 7 Pôle de Recherche Clinique, INSERM, Paris, France; 8 Normandie Univ, UNIROUEN, Department of General Practice, Rouen, France; 9 Rouen University Hospital, CIC-CRB 1404 Rouen, France; 10 University Bordeaux, INSERM, Institut Bergonié, CHU Bordeaux, CIC 1401, EUCLID/F-CRIN Clinical Trials Platform, Bordeaux, France; 11 Department of Psychiatry, Medical Faculty, University of Cologne, Cologne, Germany; 12 Universitat Autonoma de Barcelona, Barcelona, Spain; 13 Lyon Neuroscience Research Center, Inserm U1028, CNRS UMR5292, Lyon, France; 14 Normandie Univ, UNICAEN, PSL Université, EPHE, INSERM, U1077, CHU de Caen, GIP Cyceron, NIMH, Caen, France; 15 INSERM Transfert, Paris, France; 16 Minerva Health & Care Communications Ltd, Andover, United Kingdom; 17 Department of Psychology Faculty of Psychology and Educational Sciences University of Geneva, Geneva, Switzerland

**Keywords:** Dementia, Biomarkers, Anxiety, Depression

Correction to: *npj Mental Health Research* 10.1038/s44184-024-00093-8, published online 11 November 2024

In this Correction the Author Information has been updated to identify equal contributors, and joint supervisors. The original article has been corrected.

