# Supplementary material for: Sex differences in the association between repetitive negative thinking and neurofilament light
Source: Npj Ment Health Res. 2024 Nov 11;3:53. doi: 10.1038/s44184-024-00093-8 (PMC11555038; doi:10.1038/s44184-024-00093-8)
Supplement: Supplementary file 1 — Supplementary materials [file 44184_2024_93_MOESM1_ESM.pdf]

Sex Differences in the Association between Repetitive Negative Thinking, Allostatic Load and Neurofilament Light

Supplementary Materials

Table of Contents

Supplementary Material 1: Standardised cognitive tests 2
Supplementary Material 2: Allostatic load composite 3
Supplementary Material 3. Associations between RNT and each sub-domain of the allostatic load composite .. 6

Supplementary Material 1: Standardised cognitive tests

Domains evaluated	Tests	Score(s)	References	Expected performances
Global cognitive functioning	MMSE	Unique	[1]	Norms according to age, sex and education level
Executive functions	Wisconsin Card Sorting Test	Multiple	[2]	z-score > -1.65 (norms according to age, sex, and education level)
Verbal episodic memory	RL-RI16	Multiple	[3]	z-score > -1.65 (norms according to age, sex, and education level)

Supplementary Material 2: Allostatic load composite

Anthropometric category:

Body mass index (BMI) – BMI was calculated as the ratio between weight in kilograms and height in square meters.

Waist-hip-ratio (WHR) – WHR was calculated as the ratio between waist circumference in centimeters (cm) and hip circumference in cm.

Cardiovascular and respiratory category:

Systolic and diastolic blood pressure (SBP, DBP) and pulse pressure – SBP, DBP and pulse pressure were taken using an automatic blood pressure monitor (Omron, M6 Confort). Three different resting blood pressure measurements were averaged to calculate an overall SBP, DBP and pulse pressure score.

Parasympathetic nervous system functioning – The parasympathetic nervous system markers were calculated based on 2 ECG measures: a standard deviation of the average heart beat-to-beat intervals (SDANN), and root mean square of successive differences between normal heartbeats (RMSSD), recording via 2 sub-clavicular bipolar electrodes in semi recumbent position in the evening and computed by Kubios software (Kubios HRV premium 3.3.1).

Metabolic category:

Insulin – The plasma concentration of Insulin was performed by chemiluminescence assay on automated analyzer COBAS 6000 (Roche diagnostics, Meylan, France), using ready-made commercial reagent kits (Insulin ROCHE).

High and low-density cholesterol (HDL, LDL) and Triglycerides – The quantitative determination of total cholesterol, HDL cholesterol and triglycerides concentrations in serum was assessed by an enzymatic staining test in Beckman Coulter Clinical Chemistry AU

analyzers (Beckman Coulter, Villepinte, France). LDL cholesterol was calculated according to the Friedewald formula.

Creatinine – The serum creatinine concentration was assessed by an enzymatic method, with a photometric end-point reaction, performed on Beckman Coulter Clinical Chemistry AU analyzers, (Beckman Coulter, Villepinte, France).

Immune category:

C-reactive protein (CRP) and Interleukine-6 (IL-6) – The plasma concentration of CRP and IL-6 levels were performed by an ultra-sensitive electrochemiluminescence measurement technic (Meso Scale Discovery, MSD, Rockville, Marylan, USA) using V-PLEX^{TM} assays and ready-made reagent kits (V-PLEX Plus Neuroinflammation Panel 1 Human kit, K15210G, MSD).

Neuroendocrine category:

Cortisol – The serum concentration of cortisol was performed by chemiluminescence assay on automated analyzer COBAS 8000 (Roche diagnostics, Meylan, France) using ready-made commercial reagent kits (Cortisol II, ROCHE).

Dehydroepiandrosterone Sulfate (DHEA-S) – The serum concentration of DHEA-S was performed by electrochemiluminescence assay (ECLIA) on TECAN EVO (TECAN, Männedorf, Switzerland) using ready-made commercial reagent kits (DHEA-S, ROCHE).

Catecholamines – Norepinephrine (NE) and epinephrine (E) were quantified using liquid chromatography coupled to tandem mass spectrometry (LC-MS/MS). Briefly, catecholamines and their deuterated internal standards (NE-d6 and E-d6, LGC Standards GmbH,Wesel, Germany) were extracted in basic conditions using activated aluminum oxide. After elution in acid condition and evaporation, the analytes were derivatized by sodium cyanoborohydride

and acetaldehyde (Merck, Darmstadt, Germany) to form ethyl derivatives as previously reported [1,2]. Liquid chromatography was conducted on an UFLC chromatographic system (Shimadzu, Kyoto, Japan) connected to a SCIEX QTRAP® 5500 mass spectrometer (SCIEX, Toronto, Canada). Chromatographic separation was performed using a SynergiTM hydro-RP C18 column (Phenomenex, Torrance, USA) in gradient conditions, and mass spectrometry analysis was conducted using the electrospray ion source (ESI) in positive mode. Limit of quantification were <0.01 µg/L for NE and E. Recoveries were 100±7% and 96±5% in plasma samples spiked respectively with NE and E. During reproducibility assay, coefficient of variations (CV) were lower than 8.2% for NE or E at three levels of concentrations for control samples.

Supplementary Material 3. Associations between RNT and each sub-domain of the allostatic load composite

	Ruminative brooding (n = 109)		Worry (n = 110)	
	Standardised Beta (95% CI)	p-value	Standardised Beta (95% CI)	p-value
**Anthropometric category**				
Model 1	-0.06 (-0.24 to 0.13)	0.559	-0.14 (-0.32 to 0.05)	0.140
Model 2	-0.09 (-0.27 to 0.98)	0.316	-0.16 (-0.33 to 0.02)	0.076
**Cardiovascular & respiratory category**				
Model 1	-0.10 (-0.28 to 0.08)	0.273	-0.02 (-0.15 to 0.17)	0.832
Model 2	-0.11 (-0.29 to 0.07)	0.223	-0.03 (-0.22 to 0.16)	0.737
**Metabolic category**				
Model 1	0.01 (-0.19 to 0.20)	0.955	0.03 (-0.16 to 0.21)	0.780
Model 2	-0.04 (-0.21 to 0.13)	0.614	0.003 (-0.17 to 0.17)	0.969
**Immune category**				
Model 1	0.15 (-0.04 to 0.34)	0.121	0.08 (-0.11 to 0.26)	0.421
Model 2	0.16 (-0.04 to 0.35)	0.110	0.08 (-0.11 to 0.27)	0.401
**Neuroendocrine category**				
Model 1	-0.11 (-0.30 to 0.08)	0.266	0.04 (-0.15 to 0.24)	0.697
Model 2	-0.07 (-0.26 to 0.11)	0.437	0.06 (-0.12 to 0.24)	0.533

Model 1 was unadjusted. Model 2 was adjusted for age, sex and education.

References

1 Folstein MF, Folstein SE, McHugh PR. ‘Mini-mental state’. A practical method for grading the cognitive state of patients for the clinician. *J Psychiatr Res* 1975; **12**: 189–198.

2 Grant DA, Berg EA. A behavioral analysis of degree of reinforcement and ease of shifting to new responses in a Weigl-type card-sorting problem. *J Exp Psychol* 1948; **38**: 404–411.

3 Van der Linden M, Adam S, Agniel A, Baisset Mouly C. L’évaluation des troubles de la mémoire: Présentation de quatre tests de mémoire épisodique (avec leur étalonnage). *Neuropsychologie* 2004.
